# Graphene Plasmon Resonances for Electrically-Tunable Sub-Femtometer Dimensional Resolution

**DOI:** 10.3390/nano10071381

**Published:** 2020-07-15

**Authors:** Zhiyong Wu, Lei Zhang, Min Zhang, Irene Ling Li, Hong Su, Huancheng Zhao, Shuangchen Ruan, Huawei Liang

**Affiliations:** 1 Shenzhen Key Laboratory of Laser Engineering, College of Physics and Optoelectronic Engineering, Shenzhen University, Shenzhen 518060, China; 1800281004@email.szu.edu.cn (Z.W.); zhangmin@szu.edu.cn (M.Z.); liling@szu.edu.cn (I.L.L.); hsu@szu.edu.cn (H.S.); 1800281011@email.szu.edu.cn (H.Z.); scruan@szu.edu.cn (S.R.); 2 Key Laboratory for Physical Electronics and Devices of the Ministry of Education and Shanxi Key Laboratory of Information Photonic Technique, School of Electronic Science and Engineering, Xi’an Jiaotong University, Xi’an 710049, China; eiezhanglei@xjtu.edu.cn

**Keywords:** plasmon, localized surface plasmon resonances, cavity plasmon, plasmon sensor, graphene, sub-femtometer

## Abstract

A coupled graphene structure (CGS) is proposed to obtain an electrically tunable sub-femtometer (sub-fm) dimensional resolution. According to analytical and numerical investigations, the CGS can support two branches of localized surface plasmon resonances (LSPRs), which park at the dielectric spacer between two pieces of graphene. The coupled efficiencies of the odd-order modes are even four orders of magnitude higher than that of the even-order modes. In particular, a sub-fm resolution for detecting the change in the spacer thickness can be reached using the lowest order LSPR mode. The LSPR wavelength and the dimensional differential resolution can be electrically-tuned from 9.5 to 33 μm and from 4.3 to 15 nm/pm, respectively, by modifying the chemical potential of the graphene via the gate voltage. Furthermore, by replacing the graphene ribbon (GR) at the top of the CGS with multiple GRs of different widths, a resonant frequency comb in the absorption spectrum with a tunable frequency interval is generated, which can be used to detect the changes in spacer thicknesses at different locations with sub-fm resolution.

## 1. Introduction

The ultra-precise detection on extremely weak biological and physical processes occurring at the nanoscale has always been a strong desire, which can find important applications in probing DNA hybridization [1,2], molecule assembly [3,4], thermal expansion [5,6], and photomechanical effects [7,8]. On the road towards extra-high dimensional resolution, localized surface plasmon resonances (LSPRs) parking at the deep subwavelength nanocavity between coupled plasmonic nanostructures have attracted great attention [9,10,11,12]. The far-field absorption spectrum amplified by non-radiative decay of LSPRs depends intensely on the morphology, dimension, and material of the nanocavity [13,14,15,16,17]. Thus, it is possible to monitor awfully weak changes in the dimension of the nanocavity by measuring the shift of the LSPR frequency, or the equivalent LSPR wavelength. So far, various coupled plasmonic nanostructures that can support cavity plasmons have been reported, such as gold nanoparticle dimers [18], metallic nanocube on metallic film [19,20], metallic nanocube dimers [21,22,23], and gold/silver nanowire-on-mirror [24]. The dimensional resolutions of the coupled plasmonic nanostructures have reached the order of sub-picometer (sub-pm) theoretically and experimentally [23,24]. However, the sub-femtometer (sub-fm) dimensional resolution has yet to be attained, owing to the LSPR of coupled metallic nanostructures resonating at relatively short wavelengths. Moreover, the limited tunability restricted by the fixed material properties is also a stumbling block.

To tackle the aforementioned limits, a coupled graphene structure (CGS) is proposed to obtain electrically tunable sub-fm dimensional resolution, in which a graphene ribbon (GR) is tightly adhered to the upper surface of a dielectric spacer, and the lower surface of the spacer is attached to a graphene-pasted silver film. An analytical model is deduced to calculate the LSPR wavelength of the CGS. According to the calculated results, the CGS can support two branches of LSPRs parking at the dielectric spacer between two pieces of graphene, i.e., odd-order and even-order modes. The coupled efficiencies of the odd-order modes are four orders of magnitude higher than that of the even-order modes. Moreover, the coupled efficiencies of the odd-order modes decrease rapidly as the increase of mode orders. In particular, the resolution for detecting the change in thickness of the spacer can reach the sub-fm scale, which is dozens of times higher than the recently reported resolution [23,24]. The LSPR wavelength and the dimensional differential resolution (DDR) can be electrically tuned from 9.5 to 33 μm and from 4.3 to 15 nm/pm, respectively, by modifying the chemical potential of the graphene via the gate voltage. Furthermore, the sensitivity for monitoring the change in the refractive index (RI) of the spacer is 9.7 μm/RI unit (RIU), which means that the smallest change in the RI of the spacer that can be identified is on the order of 10^−6^ RIU. The greatest figure of merit (FoM) obtained can reach as high as 440, which is several times higher than previously reported values [21]. Significantly, a resonant frequency comb in the absorption spectrum with a tunable frequency interval is generated using a CGS with multiple GRs of different widths at the top. The electrically tunable sub-fm dimensional resolution facilitates the ultra-precise detection on extremely weak physical processes occurring at the nanoscale, such as thermal expansion, piezoelectric effects, and photomechanical effects.

## 2. Theoretical Model and Method

**Figure 1:**
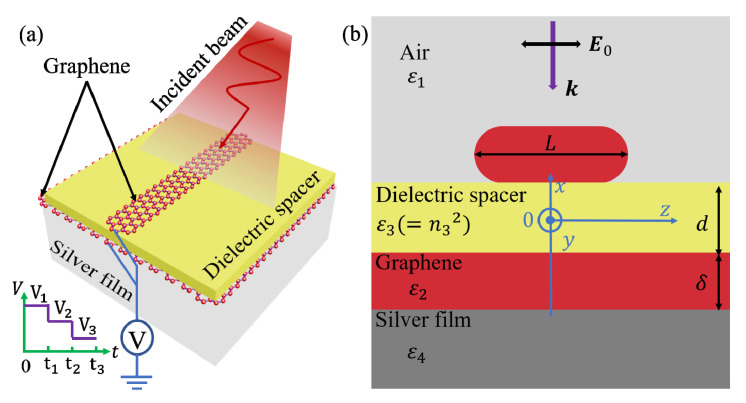
(**a**) The schematic diagram and (**b**) cross section of the coupled graphene structure (CGS). The adopted Cartesian coordinate system is shown in (**b**).

The schematic diagram of the proposed CGS is shown in Figure 1a. The lower surface of the dielectric spacer is in contact with a graphene-pasted silver film, and the upper surface is covered by another piece of GR. The chemical potential of the graphene can be actively tuned by modifying the gate voltage [25,26,27], temperature [28], chemical doping [29], electric field [30], and so on [31,32]. The cross section of the simulated structure modeled by using the commercially available software COMSOL Multiphysics (Stockholm, Sweden) is shown in Figure 1b. The entire system is placed in the air with a relative permittivity of *ε*_1_ = 1, which is illuminated by a linearly polarized plane wave from the top. The silver film is treated as a half-infinity-extended body, and its relative permittivity is modeled using the Drude model, *ε*_4_ = 1 *− ω*_p_^2^/(*ω*^2^ + *iωγ*), where *ω* is the angular frequency of the incident wave. *ω*_p_ = 9.014 eV and *γ* = 0.018 eV [33] are the plasma frequency and damping constant, respectively. To prevent unrealistically sharp edges, both ends of the GR are semicircular with a curvature, *δ*/2, where *δ* = 0.34 nm is the thickness of the graphene. The relative permittivity of the graphene, *ε*_2_, can be calculated using *ε*_2_ = *iσ*_g_/(*ωε*_0_*δ*) [34,35], where *ε*_0_ is the permittivity of vacuum. The surface conductivity of the graphene, *σ*_g_, can be evaluated using the Kubo formula as follows [28,36]:

(1) $$\sigma_{g}(\omega ,\mu_{c},\Gamma ,T)=\sigma_{\mathrm{intra}}(\omega ,\mu_{c},\Gamma ,T)+\sigma_{\mathrm{inter}}(\omega ,\mu_{c},\Gamma ,T)$$

(2) $$\sigma_{\mathrm{intra}}(\omega ,\mu_{c},\Gamma ,T)=\frac{ie^{2}k_{B}T}{\pi {ћ}^{2}(\omega +i2\Gamma )}\left\{\frac{\mu_{c}}{k_{B}T}+2\ln[\exp(-\frac{\mu_{c}}{k_{B}T})+1]\right\}$$

(3) $$\sigma_{\mathrm{inter}}(\omega ,\mu_{c},\Gamma ,T)=\int_{0}^{\infty }\frac{ie^{2}(\omega +i2\Gamma )}{\pi {ћ}^{2}}\frac{{\left[\exp(\frac{-\Omega -\mu_{c}}{k_{B}T})+1\right]}^{-1}-{\left[\exp(\frac{\Omega -\mu_{c}}{k_{B}T})+1\right]}^{-1}}{{\left(\omega +i2\Gamma \right)}^{2}-4{\left(\Omega /ћ\right)}^{2}}d\Omega$$

where *σ*_intra_ and *σ*_inter_ represent the intra-band and inter-band terms of surface conductivity, respectively. *−e* is the charge of an electron, *ћ* = *h*/(2π) is the reduced Planck’s constant, *k*_B_ is Boltzmann’s constant, and *μ*_c_ is chemical potential of graphene. The temperature and charged particle scattering rate are set as *T* = 300 K and *Г* = 0.1 meV, respectively.

When the length of the GR in the *y* direction is regarded as infinite, the CGS in Figure 1b can be reduced to a five-layer slab waveguide for surface plasmon polaritons (SPPs) propagating along the *z* direction. The SPPs are transverse magnetic (TM) modes [37], so the magnetic field component can be written as:

(4) $$H_{y}=\left\{ \begin{array}{ll} A_{1}e^{-k_{1}x}\phi & (d/2+\delta )<x\\ (A_{2}e^{k_{2}x}+B_{2}e^{-k_{2}x})\phi & d/2<x<(d/2+\delta )\\ (A_{3}e^{k_{3}x}+B_{3}e^{-k_{3}x})\phi & -d/2<x<d/2\\ (A_{4}e^{k_{2}x}+B_{4}e^{-k_{2}x})\phi & (-d/2-\delta )<x<-d/2\\ A_{5}e^{k_{4}x}\phi & x<(-d/2-\delta ) \end{array}\right.$$

where *A_j_* (*j* = 1, 2, 3, 4, or 5) and *B_l_* (*l* = 2, 3, or 4) are undetermined mode coefficients, and *d* is the thickness of the dielectric spacer. *ϕ* = *e*^−*i*(*ωt*−*βz*)^ and *k_q_*^2^ = *β*^2^ − *μ*_0_*μ_q_ε*_0_*ε_q_ω*^2^ (*q* = 1, 2, 3, or 4), respectively, where *β* is the complex propagation constant, *μ*_0_ is the permeability of vacuum, *μ_q_* = 1 for the considered nonmagnetic materials, and *ε_q_* are the relative permittivity. Based on the expression of *H_y_* and the relationship between electric and magnetic field components, the electric field components *E_x_* and *E_z_* can be solved [38]. By further employing the continuities of the tangential field components *H_y_* and *E_z_* at interfaces *x* = *d/2 + δ*, *d/2*, − *d/2*, and − *d/2* − *δ*, we obtain the following dispersion equation:

(5) $$\frac{(\frac{k_{1}}{\varepsilon_{1}}-\frac{k_{2}}{\varepsilon_{2}})\left[e^{2k_{3}d}{\left(\frac{k_{2}}{\varepsilon_{2}}-\frac{k_{3}}{\varepsilon_{3}}\right)}^{2}-{\left(\frac{k_{2}}{\varepsilon_{2}}+\frac{k_{3}}{\varepsilon_{3}}\right)}^{2}\right]+e^{2k_{2}\delta }(e^{2k_{3}d}-1)(\frac{k_{1}}{\varepsilon_{1}}+\frac{k_{2}}{\varepsilon_{2}})\left[{\left(\frac{k_{2}}{\varepsilon_{2}}\right)}^{2}-{\left(\frac{k_{3}}{\varepsilon_{3}}\right)}^{2}\right]}{e^{2k_{2}\delta }(\frac{k_{1}}{\varepsilon_{1}}+\frac{k_{2}}{\varepsilon_{2}})\left[e^{2k_{3}d}{\left(\frac{k_{2}}{\varepsilon_{2}}+\frac{k_{3}}{\varepsilon_{3}}\right)}^{2}-{\left(\frac{k_{2}}{\varepsilon_{2}}-\frac{k_{3}}{\varepsilon_{3}}\right)}^{2}\right]+(e^{2k_{3}d}-1)(\frac{k_{1}}{\varepsilon_{1}}-\frac{k_{2}}{\varepsilon_{2}})\left[{\left(\frac{k_{2}}{\varepsilon_{2}}\right)}^{2}-{\left(\frac{k_{3}}{\varepsilon_{3}}\right)}^{2}\right]}=e^{2k_{2}\delta }\frac{\frac{k_{2}}{\varepsilon_{2}}+\frac{k_{4}}{\varepsilon_{4}}}{\frac{k_{2}}{\varepsilon_{2}}-\frac{k_{4}}{\varepsilon_{4}}}$$

Owing to the finite width of the GR, electromagnetic waves are confined within the lateral range covered by the GR, thus some standing-waves with specific propagation constants can be supported by the CGS. The resonant condition of the standing-waves can be written as [23]:

(6) $$\beta =\frac{m\pi -\varphi }{L}$$

where *m* is a positive integer characterizing the number of wave-nodes in the *z* direction, and *L* is the width of the GR. *φ* = arctan[Re(*r*)/Im(*r*)] is a phase shift from scattering at the boundaries [39], where the complex reflection coefficient *r* = [(1 − *G*)/(1 + *G*)]*. The intermediate variable *G* can be written as [40,41]:

(7) $$G=\frac{1}{\lambda \sqrt{\mu_{0}/\varepsilon_{0}}}\frac{\int_{-\infty }^{\infty }-{\left(\int_{-\infty }^{\infty }E_{x}e^{-ik_{x}x}dx\right)}^{2}/\sqrt{k_{0}{}^{2}-k_{x}{}^{2}}dk_{x}}{\int_{-\infty }^{\infty }E_{x}H_{y}{}^{*}dx}$$

where *λ* is the wavelength of the incident wave and *k*_0_ = 2*π*/*λ* is the wavenumber in vacuum. By solving Equations (5) and (6), the resonance wavelength can be quantitatively determined.

## 3. Results and Discussion

**Figure 2:**
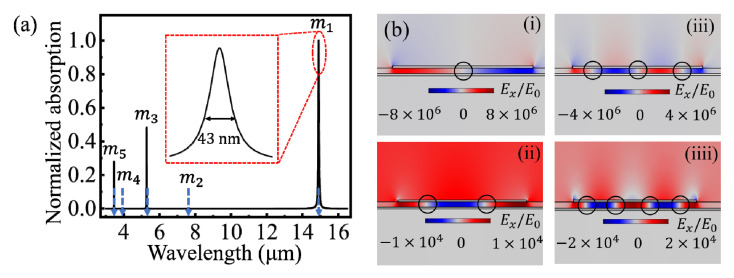
(**a**) Normalized absorption spectrum of the CGS (black line) and the positions of resonance wavelengths solved by using the analytical model (downward dashed blue arrows). The inset in (**a**) shows that the full width at half maximum (FWHM) of the *m*_1_ peak is 43 nm. (**b**) Electric field distributions corresponding to the coupled localized surface plasmon resonances (LSPRs) labeled (i) *m*_1_, (ii) *m*_2_, (iii) *m*_3_, and (iiii) *m*_4_ in (**a**). The chemical potential of graphene is *μ*_c_ = 0.5 eV. The width of GR is *L* = 40 nm. The thickness and refractive index (RI) of the dielectric spacer are *d* = 1.0 nm and *n*_3_ = 1.5, respectively.

When the wave vector of incident wave matches the oscillation frequency of free electrons of the CGS, the plasmons around the graphene are strongly coupled together at the dielectric spacer. The normalized absorption spectrum was calculated using COMSOL Multiphysics, as shown by the black line in Figure 2a. The coupled LSPRs supported by the CGS can be divided into two branches: odd-order and even-order modes. The coupled efficiencies of the even-order modes are even four orders of magnitude lower than that of the odd-order modes, so they are difficult to be observed from the absorption spectrum. The coupled efficiencies of odd-order modes decrease rapidly as the increased mode order. The positions of the resonance wavelengths calculated using the analytical model are shows by the downward dashed blue arrows in Figure 2a, which are consistent with the numerical calculation results. The first four modes labeled *m*_1_, *m*_2_, *m*_3_, and *m*_4_, correspond to electric field distributions with 1, 2, 3, and 4 zero wave-nodes at the dielectric spacer, as shown in Figure 2b. For the odd-order modes, the proportion of the electric field leaked into the free space gradually increased as the mode order increased, which corresponded to the decrease of the coupled efficiency. However, for even-order modes the percentage of the electric field diffused into the free space gradually decreased with the increase of the mode order, corresponding to the increase of coupled efficiency. Moreover, the percentage of the electric field of the even-order modes diffused into free space is much larger than that of the odd-order modes.

**Figure 3:**
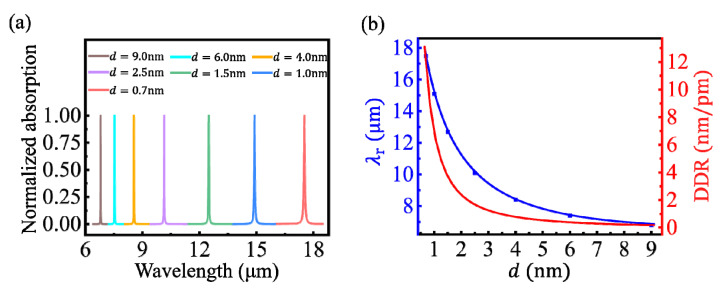
(**a**) Normalized absorption spectrums of the CGS under different spacer thicknesses. (**b**) Dependences of the LSPR wavelength (blue line) and the dimensional differential resolution (DDR; red line), respectively, on the spacer thickness for *μ*_c_ = 0.5 eV, *L* = 40 nm, and *n*_3_ = 1.5. The blue rectangles are the numerical results obtained using COMSOL Multiphysics (this scheme is also used in Figure 4, Figure 5 and Figure 6).

The coupled efficiency of the first-order mode, the lowest frequency standing wave at the dielectric spacer, was larger than that of higher-order modes. Thus, it was easier to be detected on the absorption spectrum. Moreover, the first-order mode was far away from the higher-order modes, which facilitated the monitoring of the spectral shift. Thus, our research was focused on the first-order mode hereafter. The spectral behavior of the coupled LSPR was studied in the range of the spacer thickness *d* ≥ 0.7 nm, in which the quantum tunneling effect was negligible [18,23]. As shown in Figure 3a, when *d* decreased, the LSPR wavelength, *λ*_r_, occurred a red-shift obviously, due to the increase of the electromagnetic coupled strength of the plasmons at the dielectric spacer [16,18]. Based on the proposed analytical model, the dependences of the resonance wavelength *λ*_r_ on the spacer thickness *d* are quantitatively calculated, as shown by the blue line in Figure 3b. It is observed that *λ*_r_ increased exponentially-like with the decrease of *d*, which is consistent with the previous scale behavior in other coupled plasmonic nanostructures [9,16,24]. The numerical results obtained using COMSOL Multiphysics are shown as the blue rectangles in Figure 3b, which agreed well with the analytical results. The DDR was further calculated by solving the differentiation of *λ*_r_ to *d*, i.e., DDR = |∂*λ*_r_/∂*d*|, as shown by the red line in Figure 3b. The maximum DDR is 12 nm/pm at *d* = 0.7 nm, which is dozens of times higher than the previous results of coupled metallic nanostructures [23,24]. Since the resolution limit of spectrometers used in experiments was usually 0.01 nm, a sub-fm thickness variation can be identified by monitoring the spectral shift of the first-order LSPR in CGS. Noteworthily, the resolution can be further improved by reducing the chemical potential of graphene or increasing the width of the GR.

The ultrahigh sensitivity of the LSPR in the CGS mainly results from several factors as follows. Compared with coupled metallic nanostructures, the CGS supports the LSPR resonating at a longer wavelength, which can increase electric near-field enhancement to higher orders of magnitude [42,43,44], and thus greatly improves the sensitivity. The electric near-field enhancement of the CGS beneficial for improving the sensitivity is also stronger than that of the non-coupled graphene [45,46,47]. Moreover, the CGS has a near-two-dimensional dielectric spacer, which can hold a purely coupled LSPR between two graphene, while the nanostructures with curved shapes typically contains near-degenerate plasmon modes that complicate the profile of the absorption spectrum and reduce the DDR [24].

**Figure 4:**
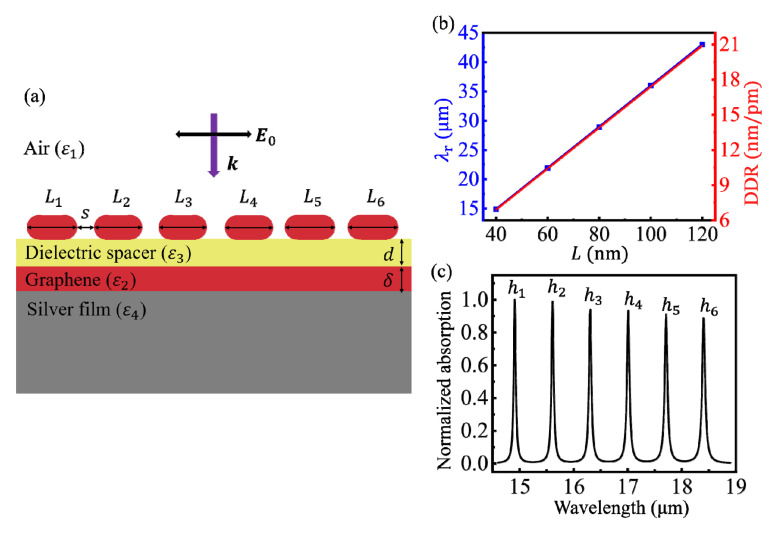
(**a**) The cross section of the CGS with a graphene ribbon array (GRA). (**b**) Dependences of the LSPR wavelength (blue line) and the DDR (red line), respectively, on the graphene ribbon (GR) width. (**c**) A frequency comb in the absorption spectrum is generated using a CGS with six GRs under *μ*_c_ = 0.5 eV, *n*_3_ = 1.5, and *d* = 1.0 nm.

The GR in the CGS is further replaced with a GR array (GRA) consisting of several GRs, as shown in Figure 4a, for obtaining more superior characteristics. According to the calculated results, when the widths of all GRs in the GRA are the same, the absorption efficiency can be greatly improved, which facilitates the observation of the resonance peaks in the absorption spectrum. When the separation between two adjacent GRs, *s*, is set as 10 nm, the coupled effect between the adjacent GRs is negligible. Thus, the absorption efficiency of resonance peaks increases linearly with the number of GRs, while the LSPR wavelength is independent on the GR number.

The resonance wavelength and the sensitivity for detecting the change in the spacer thickness were closely related to the width of the GR. The dependences of *λ*_r_ and DDR on *L* are shown by blue and red lines, respectively, in Figure 4b. The coupled LSPR can be regarded as a standing-wave mode, thus its resonance wavelength is proportional to the length of the resonant cavity, i.e., *L*. It is worth noting that the phase shift from scattering at the boundaries, *φ*, did not affect this major change trend. Moreover, the changing trends of DDR and *λ*_r_ with *L* are consistent for a given spacer thickness, which means that a larger GR width can make the sensitivity of the CGS higher.

Furthermore, multiple resonance peaks can be obtained simultaneously by using a GRA with GRs of different widths. Each resonance peak can be independently tuned by modifying the corresponding GR width. Thus, a frequency comb in the absorption spectrum can be generated by adjusting the width of each GR, as shown in Figure 4c. The wavelength intervals between adjacent resonance peaks were all 0.7 μm. The GR widths were *L*_1_ = 40 nm, *L*_2_ = 42 nm, *L*_3_ = 44 nm, *L*_4_ = 46 nm, *L*_5_ = 48 nm, and *L*_6_ = 50 nm, and the separation between adjacent GRs was *s* = 10 nm. The peaks labeled from *h*_1_ to *h*_6_ in the Figure 4c correspond to the width from *L*_1_ to *L*_6_, respectively. The wavelength interval can be tuned not only by the GR width, but also by *μ*_c_, *n*_3_, and *d*. The resonant frequency comb in the absorption spectrum can be used to detect the changes in spacer thicknesses at different locations with sub-fm resolution.

**Figure 5:**
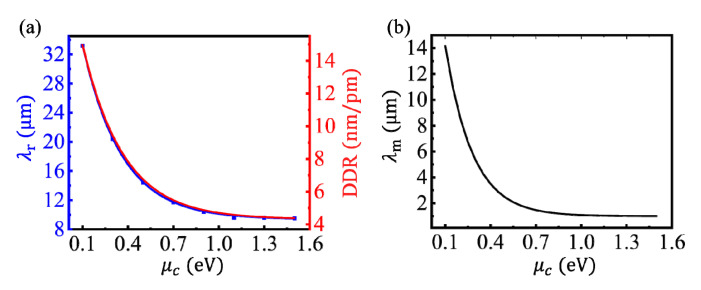
(**a**) Dependences of the LSPR wavelength (blue line) and the DDR (red line), respectively, on the chemical potential of the graphene under *L* = 40 nm, *n*_3_ = 1.5, and *d* = 1.0 nm. (**b**) The dependence of *λ*_m_ on *μ*_c_.

Different from coupled nanostructures made of noble metals, the LSPR wavelength of the CGS can be controlled by tuning the chemical potential of graphene via the gate voltage. The relationship between the chemical potential, *μ*_c_, and the voltage, *V*, can be written as $\mu_{c}=\mathrm{sgn}(n)\hbar v_{F}\sqrt{\pi \left|n\right|}$, where *v_F_* is the Fermi velocity and *n* = *C*_g_(*V* + *V*_0_)/*e* is the charge density. *C*_g_ and *V*_0_ are the gate capacitance and offset voltage, respectively [28]. The dependences of *λ*_r_ and DDR on *μ*_c_ were calculated, as shown by the blue and red lines, respectively, in Figure 5a. When *μ*_c_ decreases from 1.5 to 0.1 eV, the LSPR wavelength increased from 9.5 to 33 μm, corresponding to a res-shift, and the DDR increased from 4.3 to 15 nm/pm. The changing trends of *λ*_r_ and DDR with *μ*_c_ were highly coincident for a given spacer thickness. It is pointed out that the LSPR wavelength and the DDR can also be tuned in different ranges by changing the spacer thickness or the GR width. According to Equations (1)–(3), the relationship between *σ*_g_ and *μ*_c_ was very complicated. According to the calculations, the changing trends of *λ*_r_ and DDR were closely related to ξ=Im(ε2)/Re(ε2), so *λ*_m_ was used to describe the wavelength corresponding to the maximum value of *ξ* at a given chemical potential and the dependence of *λ*_m_ on *μ*_c_ was further calculated, as shown in Figure 5b. The changing trends of *λ*_r_ and *λ*_m_ with *μ*_c_ were very similar. However, *λ*_r_ and *λ*_m_ were not completely equal for a given *μ*_c_, because *λ*_r_ was influenced by not only the graphene, but also the structure of the CGS, such as the GR width and the spacer thickness.

**Figure 6:**
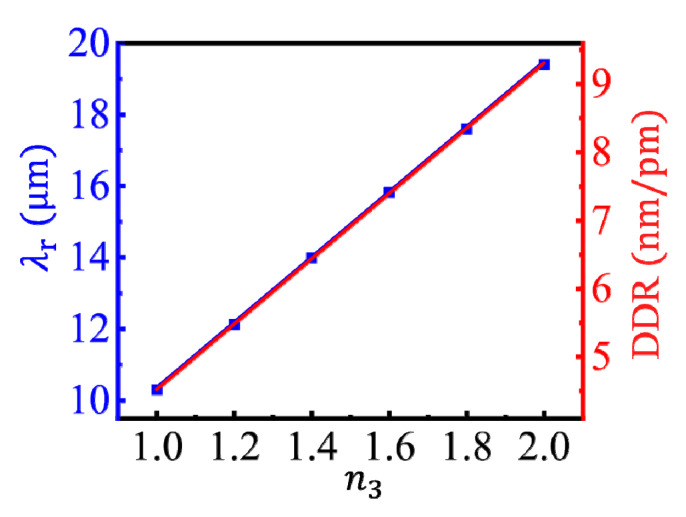
Dependences of the LSPR wavelength (blue line) and the DDR (red line), respectively, on the RI of the dielectric spacer under *μ*_c_ = 0.5 eV, *L* = 40 nm, and *d* = 1.0 nm.

Besides the dimension detection, the CGS can also be used to detect the change in the RI of the dielectric spacer, *n*_3_. As shown in Figure 6, with the increase of *n*_3_ a linear red-shift occurred in the LSPR wavelength, which is accompanied by the increase of electromagnetic coupled strength of the plasmons [16]. The gradient of *λ*_r_ to *n*_3_ was 9.7 μm/RIU, thus the smallest change in the RI of the spacer that can be identified is on the order of 10^−6^ RIU by monitoring the spectral shift of the coupled LSPR. When *n*_3_ increased from 1.0 to 2.0, the FWHM of the resonance peak increased from 22 to 74 nm. Thus the FoM, defined as the ratio of the gradient of *λ*_r_ to *n*_3_ to the FWHM, i.e., FoM = |∂*λ*_r_/∂*n*_3_|/FWHM, changed from 440 to 130, which is several times higher than the reported results of coupled metallic nanostructures [21]. Moreover, since the DDR increased linearly with the increase of *n*_3_, a dielectric spacer with a larger RI can make the dimensional resolution higher.

## 4. Conclusions

To conclude, a CGS was proposed to obtain electrically tunable sub-fm dimensional resolution. An analytical model was deduced for calculating the LSPR wavelength of the CGS. Analytical and numerical studies show that the proposed CGS could support odd-order and even-order LSPR modes parking at the dielectric spacer between two pieces of graphene, and the coupled efficiencies of the odd-order modes were even four orders of magnitude higher than that of the even-order modes. The coupled efficiencies of the odd-order modes decreased rapidly as the mode order increased. In particular, the dimensional resolution could even reach the sub-fm scale by monitoring the shift of the first-order LSPR wavelength. The LSPR wavelength and the DDR could be tuned from 9.5 to 33 μm and from 4.3 to 15 nm/pm, respectively, by modifying the chemical potential of the graphene via the gate voltage. Furthermore, the sensitivity for monitoring the change in the RI of the spacer was 9.7 μm/RIU, so the smallest identifiable change in the RI of the spacer was on the order of 10^−6^ RIU. Moreover, the greatest FoM obtained could reach as high as 440. Significantly, a resonant frequency comb in the absorption spectrum with a tunable frequency interval was generated by using a CGS with multiple GRs of different widths at the top. According to the obtained results, the proposed CGS may push the dimensional resolution of tunable plasmon sensors to a new level.
